# Consistent Partial Least Squares Path Modeling via Regularization

**DOI:** 10.3389/fpsyg.2018.00174

**Published:** 2018-02-19

**Authors:** Sunho Jung, JaeHong Park

**Affiliations:** Kyung Hee University, Seoul, South Korea

**Keywords:** consistent partial least squares, structural equation modeling, ridge-type regularization, multicollinearity, Monte Carlo simulation

## Abstract

Partial least squares (PLS) path modeling is a component-based structural equation modeling that has been adopted in social and psychological research due to its data-analytic capability and flexibility. A recent methodological advance is consistent PLS (PLSc), designed to produce consistent estimates of path coefficients in structural models involving common factors. In practice, however, PLSc may frequently encounter multicollinearity in part because it takes a strategy of estimating path coefficients based on consistent correlations among independent latent variables. PLSc has yet no remedy for this multicollinearity problem, which can cause loss of statistical power and accuracy in parameter estimation. Thus, a ridge type of regularization is incorporated into PLSc, creating a new technique called regularized PLSc. A comprehensive simulation study is conducted to evaluate the performance of regularized PLSc as compared to its non-regularized counterpart in terms of power and accuracy. The results show that our regularized PLSc is recommended for use when serious multicollinearity is present.

## Introduction

Structural equation modeling (SEM) has become a common tool in social and psychological research, including business research fields such as marketing and information systems. In no small part, this is due to its ability to provide a flexible measurement and testing framework for investigating interrelationships among observed and latent variables (Kaplan, 2009). Covariance structure analysis (CSA) (Jöreskog, 1973) and partial least squares (PLS) path modeling (Wold, 1975) represent two technically distinctive approaches to SEM (Fornell and Bookstein, 1982; Reinartz et al., 2009). Recently, a new consistent PLS estimator (PLSc) has been introduced as another alternative approach that bridges the gap between CSA and PLS (Dijkstra, 2010; Dijkstra and Henseler, 2015a). This technique rests on the idea that when PLS represents latent variables through factors, correcting for a measurement error is required to obtain consistent PLS estimates.

With the introduction of PLSc, some interest exists in evaluating its relative performance, when compared to CSA and PLS. A recent simulation study by Dijkstra and Henseler (2015b) showed that PLSc is recommended for use over traditional PLS, if the common factor model holds true for the theoretical construct. This finding is expected, given that PLSc explicitly takes the reliability of construct scores into account, and therefore, corrects the structural paths between the latent variables for attenuation, thereby enabling consistent estimates to be produced. The ability of PLSc to perform well with common factors is an important result, because SEM is frequently conducted with reflectively measured constructs.

However, Dijkstra and Henseler (2015b) clearly pointed out the potential weakness of PLSc in their simulation study, as it exhibited relatively lower statistical power and larger standard deviations under multicollinearity, as compared to other techniques. This tendency was particularly evident and problematic with small sample sizes. In practice, a high level of correlations among the latent variables is known to be quite common in the applied research (Grewal et al., 2004). In addition, because PLSc employs inter-construct correlations corrected for attenuation as input data for parameter estimations, it would likely encounter multicollinearity problems due to the possibly high correlation between independent variables. The major problem with multicollinearity is that the least squares estimators of the coefficients can produce inflated standard errors, often leading to the loss of statistical power.

Despite potential multicollinearity problems, no attempt has been made to provide methods for mitigating the problems in PLSc. Therefore, in this paper, we propose a new approach, a ridge-type of regularization, to solve multicollinearity issues in PLSc. Ridge regression (Hoerl and Kennard, 1970) is one of the possible remedies for multicollinearity in the statistical learning literature, by intentionally trading a small amount of bias for greater efficiency. Derived as an alternative to the ordinary least squares (OLS) regression estimator in the PLSc procedure, we propose a ridge least squares estimator by adding a small positive constant, called the regularization parameter, to the estimation in a straightforward manner.

The major purpose of this paper is to propose a regularized model of PLSc which handles multicollinearity problems effectively. By doing so, we believe that we can contribute to the related literature. As some researchers have already acknowledged that multicollinearity in PLSc can arouse problems in the estimation, we believe it is necessary for other researchers to consider our new approach, a ridge-type of regularization, to solve the multicollinearity issues in PLSc. The second goal of this paper is to present a comprehensive evaluation of the proposed method, relative to its non-regularized counterpart, under a variety of experimentally manipulated conditions using a Monte Carlo simulation study. With a comprehensive Monte Carlo simulation, our proposed regularized PLSc is better in dealing with a severe multicollinearity problem with common factors than ordinary PLSc.

In the next section, we discuss the previous PLSc and then propose our theoretical concept of regularized PLSc in a structural equation model. We then suggest a simulation study to confirm the newly proposed model's performance, as compared to the previous method.

## Consistent partial least squares via regularization

### A consistent reliability coefficient for PLS

Traditional PLS approximates common factors with weighted composites of observed variables. Since the composites serve as proxies for the reflective constructs, PLS construct scores are inevitably contaminated with measurement errors. Measurement errors attenuate the relationship between any two constructs, resulting in biased and inconsistent estimates of structural relationships (e.g., Bollen, 1989; Cassel et al., 2000). Correcting for measurement error attenuation would be worthwhile, as a structural equation model typically contains one or more common factors.

To achieve this purpose, Dijkstra and Henseler (2015b) have recently proposed a consistent reliability coefficient term ρ_*A*_, based on the estimation of the indicator weights under Mode A, suitable for reflective indicators. This plays a pivotal role in mitigating PLS' consistency problems in SEM with reflective measurement models. PLSc employs the coefficient of reliability to correct the latent variable correlations for attenuation, thereby adjusting the estimates to make them consistent.

The reliability measure for PLS' construct scores is determined as the squared correlation between composite scores for each latent variable and the corresponding true scores. A consistent estimator of ρ_*A*_ can be obtained so as to minimize the sums of squares of the discrepancies between the off-diagonal elements of **S** and $\hat{\Sigma }$, in which **S** is the sample covariance matrix of a latent variable's indicators and $\hat{\Sigma }$ is the implied covariance matrix based on a underlying common factor model. The coefficient of reliability can be consistently estimated using the indicator weights as follows (Dijkstra and Henseler, 2015b):

(1) $${\hat{\rho }}_{A}={\left(\hat{w}\prime \hat{w}\right)}^{2}\times \frac{\hat{w}\prime (S-\text{diag}(S))\hat{w}}{\hat{w}\prime (\hat{w}\hat{w}\prime -\text{diag}(\hat{w}\hat{w}\prime ))\hat{w}},$$

where $\hat{\text{w}}$ is the estimated weight vector for a block of indicators for the latent variable. In particular, the second part of this equation simply represents a scaling factor corresponding to the constant of proportionality between the indicator weights and the factor loadings. It plays a role in rescaling the former to the latter to adjust for an overestimation.

### Regularized consistent PLS

PLS involves two distinct models: a structural model and a measurement model. As the structural model of PLS includes a series of linear regression models for each endogenous latent variable, we begin by describing the path coefficient estimation procedures for the PLSc. The estimation procedure comprises three main steps: (1) estimate the iteratively updated indicator weights to obtain the latent variable correlations; (2) correct these correlations for attenuation using the consistent reliability estimates; and (3) perform the OLS regression to estimate the path coefficients based on the consistent construct correlations.

**Step 1:** The first step is to attempt to create latent variable proxies as linear composites of the associated observed indicators, which requires the estimation of indicator weights. This stage involves an iterative algorithm for the estimation of the weights. Accordingly, each latent variable explains as much variance as possible, with adjacent latent variables that are connected to the same latent variable. This step produces the indicator weights and correlations between the latent variable scores as inputs for the next step.

**Step 2:** Due to the presence of measurement errors, proxy correlations typically tend to underestimate the true factor correlations. As correlations among the proxies are mainly used for the estimation of the path coefficient in PLSc, a conventional attenuation correction factor can be applicable (e.g., Muchinsky, 1996). Specifically, for every pair of composite scores, a consistent construct correlation may be expressed in terms of the original proxy correlations and the two reliabilities obtained in Equation (1). That is, it is calculated by the ratio of the correlation between the construct scores to the square root of their respective reliabilities. Consequently, the correlations between the proxies associated with large measurement errors may be given greater weight than the correlations associated with smaller measurement errors.

**Step 3:** By correcting for the attenuation, due to the unreliability, as in the previous step, we are able to determine the underlying latent relationships without the distraction of measurement errors. The third step estimates the path coefficients in the structural model by means of an OLS regression. In other words, the PLSc estimator is obtained by regressing each endogenous latent variable on its causally related latent variables as follows:

(2) $$\hat{\beta }\;=\;R_{X}^{-1}r_{Xy},$$

where $\hat{\beta }$ indicates a vector of path coefficients, **R**_**X**_ is the consistent correlation matrix of the predictor variables of the structural equation, and **r**_**Xy**_ is the vector of consistent correlations between the outcome variable and the predictor variables. This illustrates that the PLSc estimator stems from the OLS regression and consistent correlation estimation.

Several variance-based SEM techniques exist, but PLSc seems to be the preferred choice of researchers for evaluating the structural model with common factors. However, although a consistent reliability coefficient helps to establish consistent estimations in the model involving factors, rather ironically, the correction for attenuation is likely to lead to a multicollinearity problem, which can give rise to spurious results. Multicollinearity is considered a major application problem in SEM, because it reduces statistical power and increases the variances for the estimated coefficients, making them unstable (Grewal et al., 2004). The more variance the coefficients have, the more difficult it is to interpret them.

To address this issue, a regularized extension of the PLSc is proposed that integrates a ridge-type of regularization into PLSc. Estimating the path parameters through regularization is straightforward. A ridge least squares estimator for $\hat{\beta }$ is given by:

(3) $$\hat{\beta }\;(\lambda )\;=\;{\left(R_{X}\;+\;\lambda I\right)}^{-1}r_{Xy},$$

where: λ denotes the regularization parameter (or tuning parameter). When λ = 0, the ridge estimates are equivalent to those obtained using ordinary PLSc (Equation 2). As with PLSc, the ridge estimator can be used as a tool for recursive models that only include unidirectional effects. The proposed regularized PLSc initially entails finding an appropriate value of the regularization parameter. It then estimates the path parameters using Equation (3), for which an optimal value of λ is included in the analysis.

A significant number of studies emphasize the practical utility of regularization in many multivariate data analysis techniques (Hastie et al., 2001; Tenenhaus and Tenenhaus, 2011; Srivastava et al., 2014). In general, the regularization parameter plays a crucial role in controlling the degree of regularization imposed on the parameters. It has the effect of shrinking the least squares estimates toward zero, thereby enabling more accurate solutions to be produced. A regularized estimator intentionally trades bias for reduction in variance. As such, it will certainly be biased (albeit slightly), but will still exhibit a much smaller variability. Therefore, the ridge estimates of parameters tend to be, on average, closer to the true population values than their least squares counterparts (see Groß, 2003, pp. 118–120). In particular, this positive effect of regularization is more pronounced under multicollinearity and/or small sample sizes (Takane and Jung, 2008).

The proposed method utilizes the *K*-fold cross-validation method to select the value of λ, which is typically a small positive constant. In the cross validation, the entire dataset is randomly divided into *K* subsets (typically, either 5 or 10). One of the *K* subsets is set aside as a validation sample, while the remaining *K*-1 subsets are used as a training sample for fitting a single structural equation model for each endogenous construct from which the estimates of the path coefficients are obtained. These resultant estimates are then applied to the validation sample to calculate the prediction error of the structural model. This procedure is repeated *k* times, changing a single group set aside systematically. The cross-validation estimate of the prediction error is accumulated over all *K* validation samples. The cross validation procedure also systematically varies the values of λ and the value that yields the lowest prediction error is finally chosen. When *K* is equal to *N* (sample size in the original data), the cross validation procedure is also known as the leaving-one-out cross validation, which appears to work reasonably well with small sample sizes (e.g., Molinaro et al., 2005).

As in its ordinary counterpart, the proposed regularized PLSc uses the bootstrap method (Efron, 1982) to estimate the standard errors of the parameter estimates. More specifically, their standard errors are calculated non-parametrically based on 5,000 bootstrap samples (Hair et al., 2011). Furthermore, the bootstrap standard errors can be used to test whether a structural parameter is statistically different from zero, based on a confidence interval approach (Aguirre-Urreta and Rönkkö, forthcoming). For instance, if the 95% confidence interval of a parameter does not include zero, then the observed effect may be considered statistically significant.

## A simulation study

The primary goal of the present simulation study is to compare the performance of the proposed regularized PLSc (hereafter referred to as RegPLSc) with that of the non-regularized PLSc. A secondary goal is to evaluate the impact of a comprehensive set of design factors and their interactions on the performance of these two estimation methods. This study builds on earlier work (Grewal et al., 2004), examining the role of multicollinearity and measurement errors on parameter recovery and inference errors in SEM. All computations for this study were carried out using MATLAB R2009a (The MathWorks, Inc.).

### Design factors

The Monte Carlo simulation involved manipulating four experimental conditions: multicollinearity (ϕ), measurement error (θ), coefficient of determination (*R*^2^), and sample size (*N*). These design factors are essentially the same as those that Grewal et al. (2004) considered in their simulations within the framework of covariance-based SEM. Prior simulation studies have shown them to be meaningful conditions in evaluating the performance of various SEM techniques (e.g., Hwang et al., 2010; Lu et al., 2011). In particular, we employed *R*^2^ as a design factor as a high *R*^2^ has the potential to improve the quality of parameter estimation in the presence of multicollinearity (e.g., Mason and Perreault, 1991; Grewal et al., 2004).

The levels of the design factors should be chosen, such that they would represent the range of values encountered in substantive studies using SEM. The selected ranges for the first three factors (ϕ, θ, *R*^2^) are basically the same as those considered in Grewal et al. (2004). First, the level of multicollinearity was varied by systematically altering the correlation between ξ_1_ and ξ_2_. The moderate condition (ϕ = 0.4) was included, plus strong (ϕ = 0.6) and extreme (ϕ = 0.8) correlation levels. The amount of random measurement error was then varied at two levels. Specifically, the composite reliability of each latent variable was set at 0.6 or 0.8 that can be considered as weak and strong, respectively. For the coefficient of determination, the value of *R*^2^ for each latent endogenous variable was set to 0.25 or 0.50, corresponding to the medium and large effect sizes, respectively, according to Fritz et al. (2012). Finally, the value of *N* was set to 30, 60, 120, or 200. These sample sizes are identical to the sizes Lu et al. (2011) considered in their simulations. Various approaches for SEM exist, but PLS path modeling has typically been recommended for use in the case of small samples (e.g., Henseler et al., 2009). Prior studies have found that PLS provides a better quality of solution in small samples (e.g., Chin and Newsted, 1999). Small sample sizes may be the rule, rather than the exception, in an empirical application of PLS (e.g., Haenlein and Kaplan, 2004).

**Figure 1:**
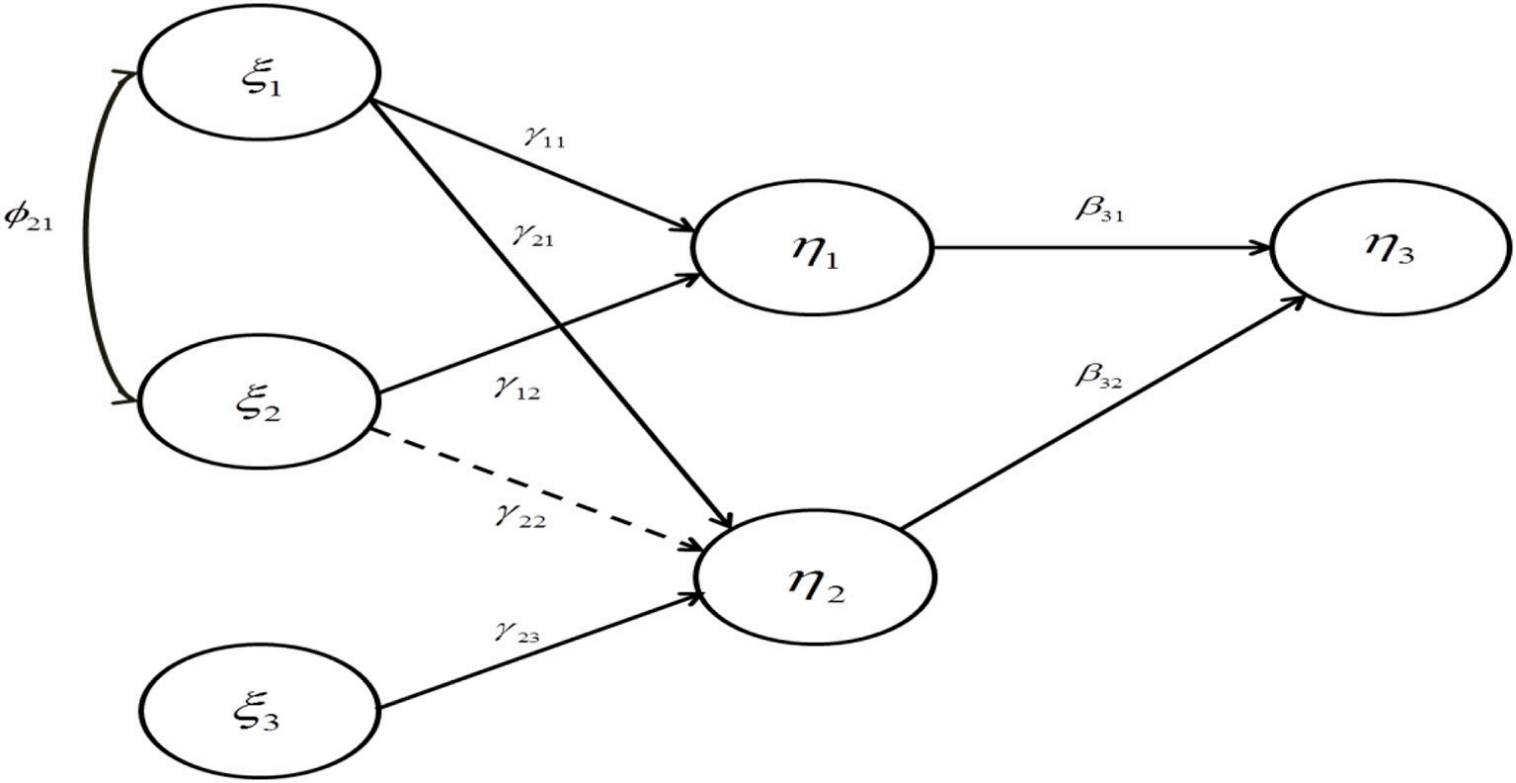
The specified model for the simulation study. Dashed line represents a path whose true value is zero.

We specified a structural equation model which consisted of six latent variables and four reflective indicators per latent variable (Figure 1). We adapted this model from Grewal et al. (2004), in which all unstandardized path coefficients were originally fixed at 0.28. Variance-based SEM, such as partial least squares, typically provides standardized parameter estimates and their standard errors. Thus, we calculated different sets of standardized parameter values based on varying levels of ϕ and *R*^2^ (Table 1).

**Table 1:** The standardized parameters.

**Collinearity**	***R*^2^**	**Population path coefficients**					
		**γ_11_**	**γ_12_**	**γ_31_**	**γ_32_**	**γ_21_**	**γ_23_**
ϕ = 0.4	*R*2 = 0.25	0.318	0.279	0.372	0.335	0.354	0.354
	*R*2 = 0.50	0.45	0.394	0.525	0.473	0.5	0.5
ϕ = 0.6	*R*2 = 0.25	0.298	0.261	0.382	0.322	0.354	0.354
	*R*2 = 0.50	0.421	0.369	0.541	0.456	0.5	0.5
ϕ = 0.8	*R*2 = 0.25	0.281	0.246	0.391	0.311	0.354	0.354
	*R*2 = 0.50	0.397	0.348	0.554	0.44	0.5	0.5

### Data generation

The full factorial design for the simulation leads to a total of 48 factor combinations (4 *Sample Sizes* × 3 *Multicollinearity* × 2 *Measurement Error* × 2 *Coefficients of Determination*). For each of the 48 different combinations, individual-level multivariate normal data were drawn from N(**0**, Σ), where Σ is the implied population covariance matrix derived from a CSA formulation using the unstandardized parameter values. During the data generation process, in some rare situations, a consistent correlation matrix is found not to be positive definite. The least squares estimator (Equation 2) fails with such a matrix. Any simulated sample was removed that failed to produce a consistent non-singular correlation matrix from further consideration to compare the two methods in an impartial manner. The first 500 replications with proper solutions were maintained for each of the combinations of the design factors.

## Simulation results

In this section, we report the ability of RegPLSc and PLSc to recover the true parameter values for the path coefficients, as well as conduct a statistical inference. The practical benefit of PLS, in empirical applications, may depend on its ability to determine the significance of a parameter estimate from the statistical power perspective. Although achieving accurate statistical inferences enables researchers to perform reliable hypothesis tests, they also put equal emphasis on the magnitude of the structural parameter to interpret the substantive significance of a result or for predictive purposes. Accordingly, evaluating the ability to recover the true parameters is important for applied researchers who would consider using PLS techniques.

### Recovery of path parameters

To assess the recovery of the parameters under the two estimation procedures, we calculated the mean absolute differences (MAD) between the parameter values and their estimates as follows:

(4) $$\text{MAD }=\;\frac{\sum_{\text{j }=\;1}^{\text{P}}\left|{\hat{\theta }}_{\text{j}}-\theta_{\text{j}}\right|}{P},$$

where: ${\hat{\theta }}_{j}$ and θ_j_ denote the parameter estimates and population parameter values, respectively, and P is the number of parameters (e.g., Mason and Perreault, 1991).

For MAD, we conducted the full-factorial five-way mixed ANOVA. A single within-subjects method factor is the estimation method (*M*, where *M* = RegPLSc or PLSc). The between-subject data factors are the above-described four experimental conditions of the study. Table 2 presents the results about the capability of the two estimation methods. As illustrated in Table 2, most of the main and interaction effects were statistically significant, due to the large number of observations, in addition to fitting all possible interactions in the ANOVA. For this reason, it is crucial to also examine the effect size (e.g., Paxton et al., 2001). Following the accepted practice for identifying a substantial effect, we will focus on the main and interaction effects, having a partial eta-squared (η^2^) greater than 2%, which deserves further examination (see Reinartz et al., 2009). According to Cohen's (1988) guidelines regarding effect sizes, a value of 0.02 represents a small effect, 0.06 a medium effect, and 0.14 or greater a large effect.

**Table 2:** The results of ANOVA test for the mean absolute differences (MAD) of parameter estimates.

	**d.f**.	**MAD**	
		***F***	**η2**
**WITHIN-SUBJECTS EFFECTS**			
*M*	1	7384.39	0.24
M^*^L	2	1351.16	**0.10**
M^*^F	1	1694.35	**0.06**
M^*^R	1	3417.41	**0.13**
M^*^N	3	1350.97	**0.15**
M^*^L^*^F	2	6.95	0.01
M^*^L^*^R	2	20.16	0.00
M^*^L^*^N	6	35.87	0.01
M^*^F^*^R	1	11.29	0.00
M^*^F^*^N	3	157.65	**0.02**
M^*^R^*^N	3	194.46	**0.03**
M^*^L^*^F^*^R	2	6.73	0.00
M^*^L^*^F^*^N	6	1.80	0.01
M^*^L^*^R^*^N	6	24.78	0.00
M^*^F^*^R^*^N	3	9.41	0.00
M^*^L^*^F^*^R^*^N	6	1.57	0.00
Error (*M*)	23952		
**BETWEEN-SUBJECTS EFFECTS**			
Intercept	1	172168.43	0.88
*L*	2	1970.69	0.14
*F*	1	52.85	0.00
*R*	1	4726.95	0.17
*N*	3	5731.48	0.42
L^*^F	2	127.31	0.01
L^*^R	2	25.51	0.00
L^*^N	6	4.57	0.00
F^*^R	1	0.57	0.00
F^*^N	3	13.43	0.00
R^*^N	3	95.12	0.01
L^*^F^*^R	2	2.24	0.00
L^*^F^*^N	6	7.17	0.00
L^*^R^*^N	6	18.32	0.00
F^*^R^*^N	3	1.44	0.00
L^*^F^*^R^*^N	6	1.45	0.00
Error	23952		

*M, method; L, multicollinearity; F, R^2^; R, reliability; N, sample size*.

*All F-values are statistically significant (p < 0.05) except for those underlined. d.f. = degrees of freedom and η^2^ = Effect Size. Interaction effects having η^2^ greater than 2% are shown in boldface*.

The analysis method (η^2^ = 0.24) had a sufficiently large main effect. This suggests meaningful differences in the average MAD between the two methods (RegPLSc = 0.14 and PLSc = 0.19). The ANOVA for MAD in the parameter recovery revealed that all two-way interaction effects were statistically significant and achieved effect sizes larger than 6%, reflecting a medium effect: Method × Multicollinearity (η^2^ = 0.10), Method × Measurement Error (η^2^ = 0.13), Method × *R*^2^ (η^2^ = 0.06), and Method × Sample Size (η^2^ = 0.15). Two additional interactions, Method × Measurement Error × Sample Size (η^2^ = 0.03) and Method × *R*^2^ × Sample Size (η^2^ = 0.02) were selected for further examination, because they were theoretically and practically related to multicollinearity problems and had an effect size above the cut-off point. We first discuss the two way interaction of Method × Multicollinearity. The remaining two-way interactions are then described below in the context of the three-way interactions that include them.

**Figure 2:**
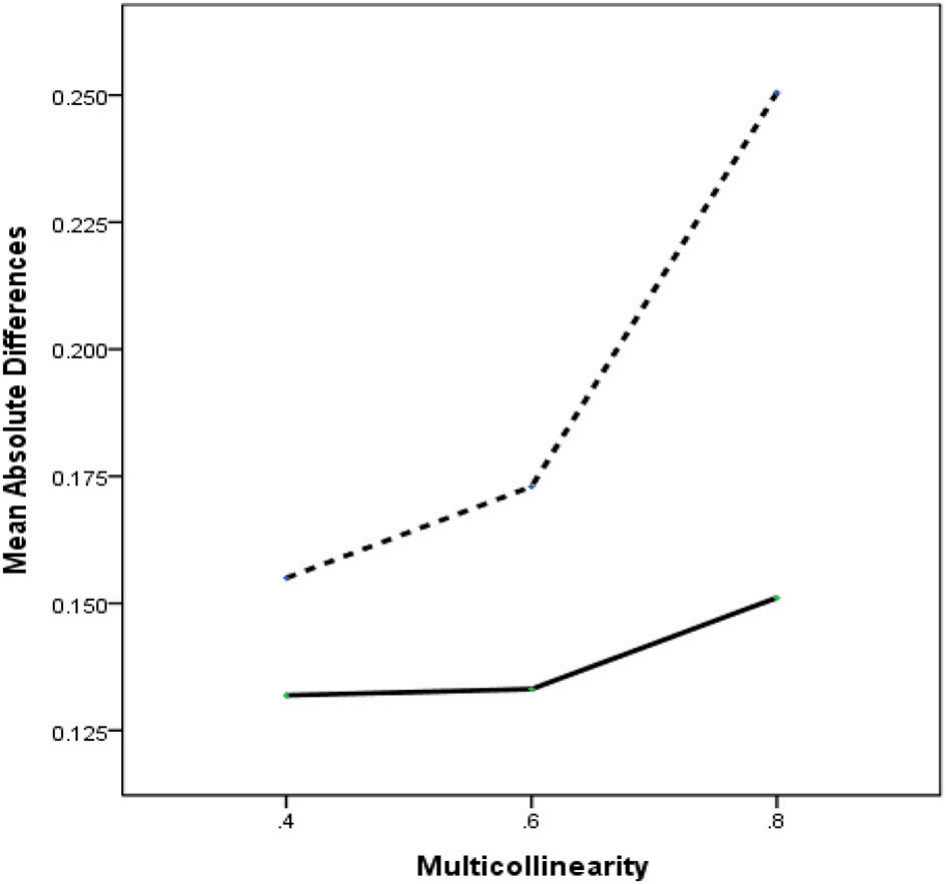
Two-way interactions of method × multicollinearity with MAD as dependent variable. Dashed line = PLSc, solid line = regularized PLSc.

Figure 2 displays the average values of MAD for each method under the three levels of multicollinearity. Overall, we could confirm that RegPLSc is notably superior across different degrees of multicollinearity. As the level of multicollinearity increases, the superiority of RegPLSc over PLSc becomes larger. A closer look at the performance of RegPLSc reveals that the method appears to be an effective tool to deal with multicollinearity in structural equation models. The average MAD value for RegPLSc under extreme conditions remains similar to that under moderate conditions. In contrast, for PLSc, such a stable tendency in the values of MAD cannot be observed, implying that PLSc is highly susceptible to multicollinearity problems.

**Figure 3:**
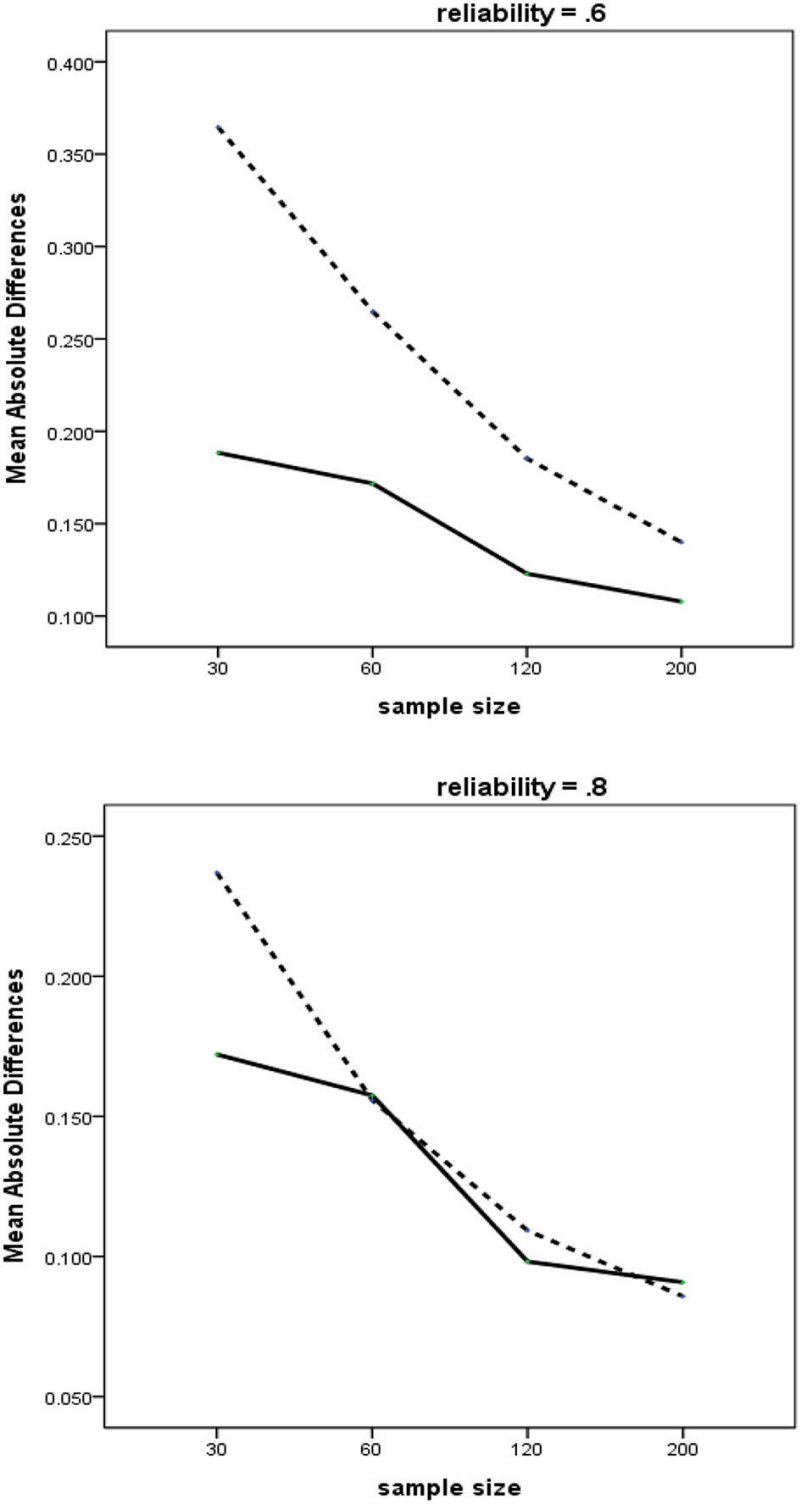
Three-way interactions of method × reliability × sample size with MAD as dependent variable. Dashed line = PLSc, solid line = regularized PLSc.

The three-way interaction of Method × Measurement Error × Sample Size is presented in Figure 3. This three-way interaction includes the two-way interaction of Method × Sample Size, which can be seen in each of the two blocks included in the figure. In general, the average MAD values for both methods tended to decrease as the sample sizes increased. We find two intriguing characteristics, depending on the level of measurement error. First, when reliability is weak, RegPLSc yields uniformly lower MAD than PLSc across all sample sizes. Second, in contrast, when measures are highly reliable, the differences in the values of MAD of the estimates become negligible, except for the smallest sample size. This implies that the adverse effects of multicollinearity may be largely offset by the measurement properties, such as reliability. The similar pattern was replicated in a simulation study by Grewal et al. (2004).

**Figure 4:**
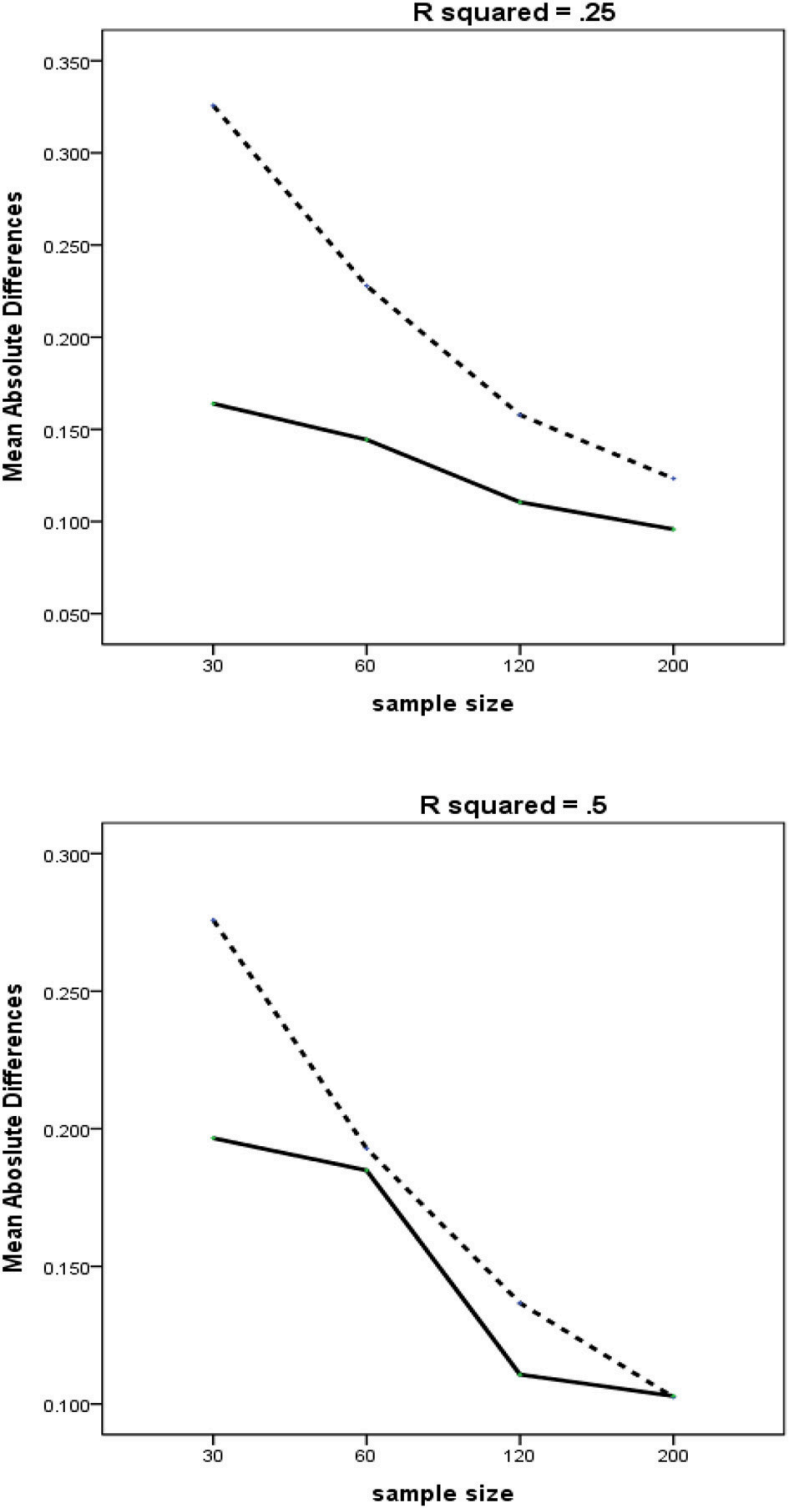
Three-way interactions of method × *R*^2^ × sample size with MAD as dependent variable. Dashed line = PLSc, solid line = regularized PLSc.

Another three-way interaction (Figure 4) is produced by the interaction of *R*^2^ with the Method and Sample Size. Our findings show that *R*^2^ is another meaningful factor that can mitigate the damaging effects of multicollinearity on the estimation accuracy. In general, PLSc and RegPLSc perform similarly for a large *R*^2^ of 0.50, while the difference becomes more markedly with a medium *R*^2^ of 0.25. Consistent with the findings of Mason and Perreault (1991), the adverse effects of multicollinearity can be markedly attenuated with a greater portion of explained variance in the dependent variable. Overall, reliability and *R*^2^ are likely to have an important impact on the good recovery of parameters in the presence of multicollinearity.

### Statistical inference

The above ANOVA test results suggest that all the experimental conditions (multicollinearity, sample size, reliability, and *R*^2^) are meaningful in differentiating the performance of the two methods in parameter recovery. To gain an additional understanding of the performance of these techniques, the statistical power was further investigated under those four experimental conditions. We estimated the standard errors of path coefficients estimates on the basis of the bootstrap method with 200 bootstrap samples (e.g., Reinartz et al., 2009).

Table 3 shows the empirically obtained statistical power of each estimation method for each combination of the experimental conditions. The numbers in the table indicate the proportion of simulation trials for which a 95% confidence interval for a path coefficient rejected the null hypothesis that path coefficient equals zero.

**Table 3:** Statistical power of PLSc and regularized PLSc.

				**γ**_**11**_			**γ**_**12**_			**γ**_**21**_			**γ**_**22**_			**γ**_**23**_			***β***_**31**_			***β***_**32**_		
				**Collinearity level**			**Collinearity level**			**Collinearity level**			**Collinearity level**			**Collinearity level**			**Collinearity level**			**Collinearity level**		
				**0.4**	**0.6**	**0.8**	**0.4**	**0.6**	**0.8**	**0.4**	**0.6**	**0.8**	**0.4**	**0.6**	**0.8**	**0.4**	**0.6**	**0.8**	**0.4**	**0.6**	**0.8**	**0.4**	**0.6**	**0.8**
*N* = 30	*R*2 = 0.25	*REL* = 0.6	PLSc	0.22	0.18	0.16	0.18	0.14	0.16	0.07	0.08	0.07	0.08	0.05	0.06	0.10	0.08	0.06	0.28	0.26	0.25	0.22	0.21	0.23
			RegPLSc	0.36	0.30	0.29	0.30	0.23	0.32	0.25	0.26	0.20	0.07	0.08	0.09	0.26	0.25	0.24	0.34	0.32	0.31	0.29	0.26	0.28
		*REL* = 0.8	PLSc	0.26	0.12	0.21	0.21	0.15	0.18	0.17	0.13	0.07	0.04	0.05	0.05	0.21	0.21	0.14	0.33	0.35	0.34	0.30	0.31	0.26
			RegPLSc	0.38	0.31	0.59	0.29	0.29	0.54	0.31	0.29	0.22	0.07	0.06	0.08	0.31	0.37	0.32	0.38	0.39	0.38	0.35	0.34	0.33
	*R*^2^ = 0.5	*REL* = 0.6	PLSc	0.29	0.23	0.15	0.23	0.16	0.15	0.10	0.06	0.09	0.08	0.04	0.09	0.10	0.07	0.11	0.10	0.07	0.11	0.36	0.29	0.32
			RegPLSc	0.49	0.48	0.41	0.40	0.37	0.40	0.41	0.35	0.29	0.07	0.09	0.11	0.41	0.38	0.42	0.51	0.56	0.58	0.47	0.44	0.47
		*REL* = 0.8	PLSc	0.46	0.32	0.15	0.41	0.27	0.17	0.35	0.20	0.18	0.05	0.02	0.05	0.42	0.28	0.21	0.60	0.65	0.64	0.59	0.49	0.49
			RegPLSc	0.63	0.64	0.52	0.57	0.55	0.52	0.54	0.54	0.52	0.07	0.06	0.09	0.57	0.57	0.57	0.72	0.75	0.76	0.68	0.64	0.67
*N* = 60	*R*^2^ = 0.25	*REL* = 0.6	PLSc	0.26	0.17	0.15	0.21	0.15	0.14	0.09	0.09	0.09	0.04	0.07	0.07	0.11	0.15	0.11	0.38	0.38	0.34	0.28	0.27	0.24
			RegPLSc	0.40	0.35	0.49	0.33	0.31	0.45	0.32	0.30	0.23	0.05	0.08	0.10	0.39	0.39	0.36	0.44	0.43	0.41	0.37	0.33	0.35
		*REL* = 0.8	PLSc	0.42	0.34	0.33	0.39	0.26	0.24	0.41	0.32	0.15	0.05	0.04	0.04	0.51	0.51	0.46	0.61	0.63	0.60	0.53	0.49	0.47
			RegPLSc	0.53	0.53	0.84	0.51	0.51	0.80	0.48	0.45	0.36	0.06	0.05	0.10	0.53	0.53	0.52	0.58	0.61	0.60	0.52	0.51	0.49
	*R*^2^ = 0.5	*REL* = 0.6	PLSc	0.41	0.29	0.15	0.38	0.28	0.14	0.18	0.12	0.07	0.05	0.05	0.05	0.24	0.21	0.12	0.58	0.58	0.61	0.53	0.51	0.48
			RegPLSc	0.62	0.66	0.58	0.60	0.61	0.55	0.58	0.48	0.40	0.08	0.09	0.11	0.58	0.57	0.59	0.70	0.75	0.76	0.68	0.69	0.62
		*REL* = 0.8	PLSc	0.77	0.57	0.24	0.67	0.53	0.22	0.81	0.60	0.26	0.05	0.04	0.06	0.87	0.88	0.72	0.90	0.91	0.92	0.83	0.80	0.80
			RegPLSc	0.89	0.84	0.74	0.82	0.81	0.72	0.79	0.71	0.70	0.07	0.11	0.11	0.81	0.86	0.86	0.94	0.94	0.94	0.88	0.87	0.88
*N* = 120	*R*^2^ = 0.25	*REL* = 0.6	PLSc	0.38	0.23	0.19	0.26	0.20	0.18	0.28	0.17	0.10	0.04	0.05	0.06	0.36	0.33	0.24	0.56	0.57	0.56	0.46	0.47	0.42
			RegPLSc	0.54	0.45	0.55	0.40	0.36	0.54	0.42	0.31	0.28	0.05	0.05	0.07	0.53	0.53	0.57	0.63	0.63	0.64	0.55	0.53	0.53
		*REL* = 0.8	PLSc	0.74	0.54	0.58	0.68	0.48	0.41	0.81	0.64	0.30	0.06	0.04	0.04	0.85	0.86	0.85	0.91	0.93	0.91	0.85	0.85	0.69
			RegPLSc	0.81	0.74	0.97	0.79	0.66	0.94	0.83	0.76	0.58	0.06	0.07	0.09	0.87	0.89	0.90	0.92	0.94	0.92	0.86	0.87	0.77
	*R*^2^ = 0.5	*REL* = 0.6	PLSc	0.66	0.44	0.16	0.60	0.40	0.15	0.60	0.38	0.15	0.04	0.04	0.08	0.71	0.62	0.38	0.83	0.84	0.88	0.78	0.73	0.63
			RegPLSc	0.84	0.73	0.56	0.77	0.70	0.55	0.79	0.69	0.47	0.04	0.08	0.10	0.85	0.85	0.86	0.92	0.94	0.93	0.89	0.87	0.81
		REL = 0.8	PLSc	0.98	0.86	0.51	0.93	0.79	0.33	0.99	0.96	0.60	0.06	0.05	0.03	0.99	1.00	0.97	1.00	1.00	1.00	0.98	0.98	0.97
			RegPLSc	1.00	0.98	0.94	0.97	0.97	0.85	0.99	0.99	0.88	0.09	0.11	0.15	0.99	1.00	0.99	1.00	1.00	1.00	1.00	1.00	0.99
*N* = 200	*R*2 = 0.25	*REL* = 0.6	PLSc	0.60	0.35	0.25	0.51	0.32	0.23	0.55	0.36	0.16	0.04	0.04	0.06	0.68	0.68	0.55	0.80	0.83	0.81	0.70	0.70	0.57
			RegPLSc	0.73	0.56	0.70	0.65	0.53	0.71	0.66	0.55	0.34	0.02	0.07	0.09	0.75	0.80	0.77	0.84	0.86	0.87	0.75	0.76	0.68
		*REL* = 0.8	PLSc	0.94	0.73	0.75	0.85	0.69	0.63	0.95	0.87	0.50	0.05	0.04	0.06	0.97	0.98	0.99	0.99	1.00	0.94	0.97	0.97	0.88
			RegPLSc	0.97	0.92	1.00	0.93	0.88	1.00	0.96	0.93	0.78	0.07	0.10	0.16	0.98	0.98	0.99	0.99	1.00	0.93	0.99	0.98	0.95
	*R*^2^ = 0.5	*REL* = 0.6	PLSc	0.85	0.63	0.21	0.81	0.54	0.19	0.92	0.70	0.25	0.04	0.04	0.04	0.96	0.94	0.72	0.97	0.97	0.98	0.94	0.92	0.90
			RegPLSc	0.96	0.91	0.76	0.92	0.89	0.72	0.97	0.91	0.70	0.05	0.10	0.11	0.98	0.98	0.96	0.99	0.99	1.00	0.99	0.99	0.97
		*REL* = 0.8	PLSc	1.00	0.97	0.65	1.00	0.93	0.56	1.00	1.00	0.82	0.06	0.06	0.04	1.00	1.00	1.00	1.00	1.00	1.00	1.00	1.00	1.00
			RegPLSc	1.00	1.00	0.99	1.00	1.00	0.98	1.00	1.00	1.00	0.10	0.12	0.16	1.00	1.00	1.00	1.00	1.00	1.00	1.00	1.00	1.00

*REL, reliability; R^2^, explained variance; N, sample size; PLSc, consistent PLS; RegPLSc, regularized PLSc*.

The results suggest that under multicollinearity, RegPLSc has an advantage over PLSc with respect to detecting statistical significance, given that the hypothesized effect actually exists in the population. This pattern of results replicates the findings for path coefficient estimation accuracy. RegPLSc can maintain very similar levels of statistical power, regardless of the degrees of multicollinearity, whereas PLSc is highly hampered by severe multicollinearity. For RegPLSc, it is apparent that the statistical power varies as a function of the sample size, reliability, and *R*^2^. More specifically, the minimum reasonable size of the sample (*N* = 30) in this particular study can lead to unacceptably low levels of statistical power. However, even under extreme multicollinearity, a small sample size of *N* = 60 is adequate for satisfactory statistical power (close to or above 80%), if *R*^2^ is large and reliability is high. With the larger sample sizes, RegPLSc is able to achieve appropriate statistical power (above 80%) in almost all cases, if reflective measures are highly reliable. This highlights the importance of reliable measurements in the presence of multicollinearity. Conversely, when multicollinearity is extreme, PLSc still fails to achieve a sufficient statistical power for γ_11_ and γ_12_, which are substantially affected by high correlations between ξ_1_ and ξ_2_, even if reliability is high, *R*^2^ is large, and the sample size is relatively large (*N* = 200).

Although researchers often pay more attention to the control of Type II error for theory testing in the SEM literature, they also need to consider whether an estimation method shows good control of Type I error rate (e.g., α = 0.05). Variance-based SEM techniques sometimes tends to favor less parsimonious models as they might fail to control Type I error rate (e.g., Henseler, 2012; Dijkstra and Henseler, 2015b). In general, a ridge type estimator produces more stable estimates of parameters, for which we have to pay with bias, making them prone to inflated Type I errors (Erickson, 1981). It is therefore important to evaluate the ability of RegPLSc to control the Type I error rate under multicollinearity. For the effect γ_22_ = 0, PLSc maintained an overall Type I error rate of 5%. This result is in agreement with simulation results already obtained by Dijkstra and Henseler (2015b). Although RegPLSc seems to maintain marginally acceptable levels of Type I error (average = 0.085, minimum = 0.02, maximum = 0.164), Table 3 suggests that it can have inflated Type I error rates, even in relatively large samples, in the case of severe multicollinearity. PLSc adequately controls Type I error under all conditions, whereas RegPLSc provides greater power. If prior research and theory are sufficient to hypothesize structural model relationships, then we recommend using RegPLSc for theory testing.

## Conclusions

A recently developed PLSc is regarded as a viable alternative to traditional PLS if the common factor model holds true. However, in practice, PLSc may suffer from multicollinearity. In this paper, PLSc was combined with ridge-type regularization in order to deal with potential multicollinearity problems. The ridge least squares estimates of the path coefficients can be found by adding the regularization parameter into the OLS estimation. The optimal value of the parameter may be chosen through cross-validation. Our overall conclusion is that the proposed regularized PLSc is successful while dealing with a severe multicollinearity problem in structural equation models with common factors.

A comprehensive Monte Carlo study was conducted which systematically compared the relative performance of the regularized PLSc with non-regularized PLSc in the presence of multicollinearity. In so doing, it provides a greater understanding of the capability of these two estimation methods in terms of parameter recovery and inference errors. The primary goal of this section is to briefly discuss the implications of the simulation study and provide guidelines for choosing between the two methods.

The following summarizes the major findings for each performance measure.

1. Mean absolute differences (MAD): both methods behave similarly in terms of MAD under moderate multicollinearity. If multicollinearity is from strong to extreme, the regularized PLSc generally recovers the path coefficients much better than non-regularized PLSc. The superior parameter recovery of the regularized PLSc over its non-regularized counterpart is found in most sample sizes considered, particularly when reliability is weak or when *R*^2^ is moderate. When the sample size is very small (*N* = 30), the regularized PLSc has smaller MAD than the ordinary PLSc, regardless of the levels of reliability and *R*^2^.
2. Power: as long as multicollinearity is around 0.4, reliability is high, and the sample size is more than 100, researchers should not be overly concerned about the estimation accuracy and statistical power of PLSc. However, if a higher level of multicollinearity is present in the data, the regularized PLSc should be the preferred choice of researchers. Under high or extreme multicollinearity, it has the adequate statistical power, even with relatively small sample sizes, as long as the reliability is high.

These findings have important implications for researchers who use PLS path modeling to inform substantive hypotheses. First, if researchers are ensured that no serious multicollinearity is present, there may be little reason to choose the regularized PLSc over the non-regularized PLSc, since PLSc generally resulted in similarly accurate parameter estimates and reliable statistical inference. However, this is true only when a measure is reasonably reliable. Otherwise, our results suggest that the regularized PLSc should be the method of choice. Second, when assessing structural models under conditions of multicollinearity, the regularized PLSc is highly recommended for use over non-regularized PLSc in all situations involving sample size, reliability, and *R*^2^.

Despite these important contributions, the present study has a few limitations. First, this study was designed to generate synthetic data within a continuous variable framework. Covariance structural models are often fitted to the data measured on ordinal categorical scales.

Thus, it might be interesting to investigate the relative performance of ordinal PLSc (Schuberth et al., 2018) vs. its regularized extension with the sample matrix of ordinal-scale variables. More methodological work is needed on how to accommodate ordinal data within the framework of regularized PLSc. Second, as is the case with all Monte Carlo simulation studies, the relative performance of each method is conditioned on the specific levels chosen for the experimental conditions. Although the current simulation took into account important experimental conditions frequently used in Monte Carlo simulation studies within the framework of SEM, it is necessary to contemplate a wider range of models and conditions for more careful investigations of the relative performance of the two approaches in future research.

## Author contributions

SJ contributed to conducing all research activities including technical development, empirical analyses, and manuscript writing; JP contributed to technical development and manuscript writing.

### Conflict of interest statement

The authors declare that the research was conducted in the absence of any commercial or financial relationships that could be construed as a potential conflict of interest.
